# Cranial Surgery

**Published:** 1889-01

**Authors:** De Saussure Ford

**Affiliations:** Augusta, Ga.


### CRANIAL SURGERY.*

BY DE SAUSSURE FORD, M. D., AUGUSTA, GA.

New impulse has been given recently to brain surgery, invited by the distinguished Ferrer and later Horsley, in their efforts to localize in the brain motor areas, and now it has been demonstrated by many surgeons that it is not so dangerous to use the trephine, nor does death ensue from the loss of cerebral material.

In March, 1861, as reported in the *Southern Medical and Surgical Journal*, a man was stricken by a large rock, comminuting the left lateral half of the frontal bone, which required me to use

*Read before the Southern Surgical and Gynecological Association.

the trephine in two places to relieve the compressed brain. After the bones were removed the membranes were discovered torn, and two tablespoonfuls of brain substance were collected—the man recovered without any trouble more than slight suppuration for a week or ten days, and has been ever since, and is to-day, a perfectly sound man without any brain aberration of any kind.

Recently I have removed a part of the occipital bone, including the groove for the right lateral sinus which was not injured, which pieces are here presented for inspection.

September 6, 1888—W. S., German boy, nine years old, sustained comminuted fracture of left leg, middle third, and comminuted fracture of left side of occipital bone, by being thrown from the railroad track by the cowcatcher of engine. Saw him an hour after injury—had not reacted, unconscious, pupils contracted, pulse small and frequent. Put up leg in felt splints temporarily. There was a small lacerated wound of scalp admitting end of little finger, which discovered fracture—sent for instruments and assistance. A flap in scalp, including pericranium, leaving wound in its centre, was dissected up, disclosing the character of fracture; the bones are presented for inspection, and it will be noted that a considerable extent of the groove for the left lateral sinus has been taken from the skull. Ordinary antisepsis was used; parts irrigated with 1-3000 bichlor. and instruments taken from carbolic acid solution; but the lateral sinus was teased out of the groove with my finger-nail, which had not been toiletted by nail brush or even pen-knife. These particles of bones were taken out principally with my fingers. There was no wound in the *dura mater*. The flap was sutured with ordinary silk, no drainage tube used and the wound dressed with absorbent cotton saturated in bichloride solution. The operation performed in the forenoon, two hours after injury; that afternoon I saw him and found him still unconscious, and that night at 8 p. m., just as I entered, he sustained his first convulsion, which was violent, lasting ten minutes, followed by tracheal râles, with failing pulse, and I announced to his family that he was dying. I turned him over on his belly, holding his head over the side of the bed, when an abundant watery fluid flowed from mouth and nostrils, relieving his lungs so conspicuously that I turned him back to the bed on his right side. Within two hours his respiration became less embarrassed, and his pulse, feeble and frequent, continued from 142 to 140 until the following Monday, 9:30 a. m., forty-eight hours after injury. Temperature never above 102°, nor respiration above 30 after operation. Bromide, chloral, hyoscamus and digitalis were used to quiet his restlessness, and from fifteen to twenty grains bisulph. quinine taken daily for ten days—milk diet, absolute quiet in dark room. The first dressing was removed four days after operation, when slight suppuration of tuft of pericranium which protruded through original scalp laceration. He went on to good recovery without any yet manifest disturbance in intellect or dispositon, and is walking on his crutches. The case illustrates the value of early inspection of cranial fracture, and the prompt removal of loose bones, anticipating acute encephalitis and possible after epileptiform seizures, and proves that this region can be successfully even trepanned without necessarily injuring the lateral sinus. More detailed history of daily course and conduct of the case will be given, if the Society will so allow in our published transactions.

TREPANNING TWICE ON EACH SIDE OF LONGITUDINAL SINUS, ONE INCH POSTERIOR TO CORONAL SUTURE, WITH REMOVAL OF INTERMEDIATE BONE DIRECTLY OVER THE SINUS WITHOUT INJURY TO IT.

L. B.—White, female, age 12, was brought to me for surgical advice. The previous history of the case is forwarded with this resumé of my work.

November 28th, 1888, (last Wednesday) the patient was carried to the amphi-theatre of the Medical College of Georgia, and with the assistance of Dr. Holland, her physician, and Professors Geddings, Wright, Lamb, and other of my associates, after administering two ounces whiskey, she was chloroformed. (I use chloroform with children and parturient women, but ether with adults in surgical work). An incision was made through scalp and pericranium, leaving depressed old cicatrice, in its centre—this flap of sufficient breadth and length to use the trephine on both sides of sagittal suture, which was done, the intervening bone was taken from over the longitudinal sinus without wounding it, and the dura mater was not injured either by the Hay's saw or trephine. The students of the medical class, before whom the operation was performed, were allowed to view the blue sinus intact. To the touch the membrane was thickened, and the brain below *seemed* hardened, and had I not been afraid (I frankly confess it) my judgment prompted me to ligate the sinus in two places, and incise the part between with the dura mater, and possibly go further and incise a portion of the cortical substance of the brain in that region, which seems to be, according to Horsley and others, the motor area which would explain the motor symptoms presenting in this child's trouble. I am most anxious to relieve the child, but if the recent operation does not suffice, am I warranted in a future effort to open the dura and attack the cortical substance as I have indicated, and as cases of others have been followed with good results. The specimens of the bone removed are for inspection, when will be remarked the unusual thickness of the skull for a child of only twelve years of age. She passed through a two hours' ordeal very well, and after the flap was sutured with ordinary silk, and bichloride dressing applied, she was conveyed to her boarding-house, where she rested well that afternoon and night after repeated doses of whiskey by Dr. Holland to bring about reaction. I ordered three grains bisulph. quinine three times a day, to commence the next morning, and milk and egg-nog for diet and ice water to drink, and 1-32 grain morphia, if restless, and apparently in pain. She did not require the morphia and has not taken an opiate up to this time—three days after operation, Saturday evening.

November 27th, 9 a. m., temp. 102; 9 p. m., temp. 103. During day *moved herself* in bed, with slight assistance. Has taken quinine and nourishment.

November 30, 9 a. m., temp. 99.2; 9 p. m. temp. 101.

December 1st, 9 a. m., temp. 99.2; 9 p. m., temp. normal.

Bandages became disarranged, dressed wound with absorbent cotton and carbolic acid solution. No suppuration. She took one pill of podophyllin gr. ⅛, ext. nux vom. gr. ⅛, ext. hyosciami, gr. ¼, ext. belladonna gr. ⅛ at 9 p. m. yesterday, November 30th, but not operating by 9 a. m., this December 1, she took two of the same pills, followed 4 p. m. December 1st by enema of warm soap-suds, resulting in large action from bowels. Her habit has been that of constipation and she had not been relieved for four days before the operation, but it being her habit, I did not consider it important to delay a day to "prepare her system." Since movement from bowels, she has flexed her thighs upon abdomen the first time since last attack in September of this year, and to-night the doctor reports that she kicked the cover from her feet and legs with decided force. I have noticed, even thus early, a marked diminution in the clonic movements of the superior extremities, being certain that "the wish is not father to the thought."

After reading the article by Horsley, "On Topography of the Cerebral Cortex," in *American Journal Medical Sciences*, April, 1887–342 *et seq.*, I am disposed to believe, with his experience and skill, my patient would have proved one of great interest, as verifying brain localization as far as motor disturbances are involved. I trust that this short resume of this recent case, still under observation, may invite full, frank, free canvass by the Society, some of whom may encourage my study in this field of surgery.

